# IsletSwipe, a mobile platform for expert opinion exchange on islet graft images

**DOI:** 10.1080/19382014.2023.2189873

**Published:** 2023-03-29

**Authors:** David Habart, Adam Koza, Ivan Leontovyc, Lucie Kosinova, Zuzana Berkova, Jan Kriz, Klara Zacharovova, Bas Brinkhof, Dirk-Jan Cornelissen, Nicholas Magrane, Katerina Bittenglova, Martin Capek, Jan Valecka, Alena Habartova, František Saudek

**Affiliations:** a Laboratory of Pancreatic Islets, Center of Experimental Medicine, Institute for Clinical and Experimental Medicine (IKEM), Prague, Czech Republic; b Dino School & Novy PORG, Prague, Czech Republic; c Diabetes Center, Institute for Clinical and Experimental Medicine, Prague, Czech Republic; d Department of Internal Medicine, Leiden University Medical Center (LUMC), Leiden, Netheralnds; e Nuffield department of surgical sciences, Oxford Consortium for Islet transplantation, Oxford, UK; f Light Microscopy Laboratory, Institute of Molecular Genetics of the Czech Academy of Sciences, Prague, Czech Republic; g Laboratory of Biomathematics, Institute of Physiology of the Czech Academy of Sciences, Prague, Czech Republic; h Redox Photochemistry Lab, Institute of Organic Chemistry and Biochemistry of the Czech Academy of Sciences, Prague, Czech Republic

**Keywords:** Consensus building, deep learning, expert opinion exchange, ground truth, human islets, image annotation, islet counting, mobile application, islet graft quality control, islet isolation, islet transplantation, user experience

## Abstract

We previously developed a deep learning-based web service (IsletNet) for an automated counting of isolated pancreatic islets. The neural network training is limited by the absent consensus on the ground truth annotations. Here, we present a platform (IsletSwipe) for an exchange of graphical opinions among experts to facilitate the consensus formation. The platform consists of a web interface and a mobile application. In a small pilot study, we demonstrate the functionalities and the use case scenarios of the platform. Nine experts from three centers validated the drawing tools, tested precision and consistency of the expert contour drawing, and evaluated user experience. Eight experts from two centers proceeded to evaluate additional images to demonstrate the following two use case scenarios. The Validation scenario involves an automated selection of images and islets for the expert scrutiny. It is scalable (more experts, images, and islets may readily be added) and can be applied to independent validation of islet contours from various sources. The Inquiry scenario serves the ground truth generating expert in seeking assistance from peers to achieve consensus on challenging cases during the preparation for IsletNet training. This scenario is limited to a small number of manually selected images and islets. The experts gained an opportunity to influence IsletNet training and to compare other experts’ opinions with their own. The ground truth-generating expert obtained feedback for future IsletNet training. IsletSwipe is a suitable tool for the consensus finding. Experts from additional centers are welcome to participate.

## GRAPHICAL ABSTRACT

**Figure:**
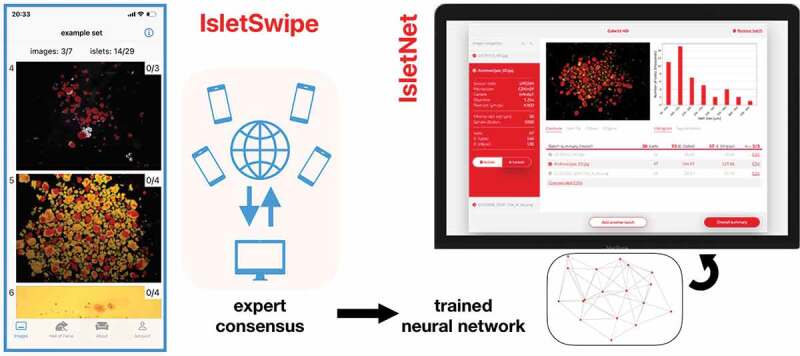

## Introduction

For decades, the clinical transplantation of isolated pancreatic islets has been the pioneering method for cell-based approach to diabetes cure.^1,2^ Pancreatic islets are isolated from cadaver pancreas (for allogeneic transplantation) or from a resected part of pancreas (for autologous transplantation).^3^ The clinical islet isolation^4,5^ is completed with the graft quality control step.^6,7^ The quantification of isolated pancreatic islets in terms of the number, volume, size distribution, and purity (islets are contaminated with the remaining exocrine tissue). Graft samples are taken and rapidly stained with dithizone^8^ to distinguish the insulin containing islets from the exocrine tissue using light microscopy.

Since 1990, the standard method for the islet enumeration is the fully manual counting under a microscope equipped with an eyepiece reticle. The individual islets are sorted into size categories by 50 µm diameter increments and multiplied by the respective size-to-volume conversion factors, to calculate the total number of islet equivalent units.^9^ Digital microscopic images are then acquired for documentation purposes only.

Alternatively, the digital images are acquired for the sake of islet counting using an analytical software. Several approaches have been developed but did not spread among many centers.^10–20^ In addition, the often employed thresholding step in the image segmentation affecting the sorting of islets into size categories is operator-sensitive, thus introducing an inadvertent subjectivity. The current computer-assisted methods for quantifying the isolated islets in the graft are labor-intensive, low throughput, subjective, and rather locally used.

Addressing these issues, we previously developed a generally available deep learning-based web service IsletNet for fully automated analysis of a wide range of locally produced microscopic images of dithizone-stained islets, generating a final report.^21^ The current IsletNet neural network was trained using images from numerous centers.^22,23^. All annotations for the IsletNet training were produced by a single ground truth generating expert. Given the inherent subjectivity of the islet image interpretation (particularly in the case of embedded, adjacent, or poorly stained islets), finding an expert consensus for the training and enabling external validation of the ground truth from other centers is needed but so far very limited due to the lack of a suitable opinion exchange tool.

Here, we describe the first communication platform for collecting expert opinions on the exact positions of islet contours in the microscopic images of dithizone-stained isolated pancreatic islets. Islet experts from three independent centers participated in this pilot study designed to demonstrate the use case and the feasibility of the novel platform.

## Material and methods

### IsletSwipe programming

The system comprises a web interface with an analytical console and a mobile application. A central API, housed by the PHP Symfony-built web interface, connects the components, and facilitates their secure communication. The mobile application was developed using the platform-independent language Dart and the framework Flutter which handles the processes of capturing user gestures and enables manipulation with islet images. All data is synchronized with the web application on mobile app startup and is stored in a local SQLite-based database which is also used for writing user ratings on images, islets, and user-drawn contours. The web interface makes use of ReactJS frontend elements, as well as a direct connection to the database using Doctrine, to modify or read islet images, users, or statistical data. An analytical console, which is called through the Symfony Process class, creates contour images by isolating the edges of islets on black and white masks and overlaying them onto the original islet images.

### Analysis of experts’opinions

Three types of expert opinions are collected using IsletSwipe application and then exported from IsletSwipe web interface for a subsequent analysis.

The qualitative classifications of islet images and marked islets were exported in csv format and then analyzed using Numbers (v10.1, Apple).

The experts’ annotations (islet separating lines and contours) together with the red template lines were exported in png format and then analyzed using a series of macros in Fiji,^24^ My-segmentation tool in IsletNet,^23^ and a web-tool for generation of box plots.^25,26^ The macros were created in IJM (ImageJ Macro Language) for Fiji software package (fiji.sc) to automatically processes all lines and images in a given folder.

The islet separating line (1 px width) was first trimmed to the islet contour of the ground truth segmentation mask. In the next step, the trimmed expert’s line (black) was compared to the trimmed reference line (red) by measuring the distances between the corresponding points of the respective lines and then computing the average distance between the red and black line. The average distance was taken as the measure of expert’s line drawing accuracy and was considered good if < 2 px.

The full islet contours (black and red) were first converted to 8-bit masks. The areas encircled by the red and black lines were then converted into the islet volumes (V) using the current standard spherical model.^9^ The relative error (RE) was calculated as RE = Abs(V_expert_ - V_template_)/V_template_.

The spread of the individual values was analyzed using the box plot.

The coefficient of variation (CV) calculated from the expert’s volume triplicates was used as the measure the expert’s repeatability.

### Microscopic images

Human pancreatic islets were isolated at IKEM and LUMC, stained with dithizone by local methods. The microscopic images of the islet graft samples were acquired using local microscopic techniques. The resulting images had the dimensions 2048 × 1536 px or 2850 × 1540 px and the pixel size ranged from 1.41 to 3.77 µm/px.

The images were manually annotated by the ground truth-generating expert, using the Free select tool combined with a macro in the image editor GNU Image Manipulation Program for macOS (GIMP v2.8, https://gimp.org). Grey scale masks distinguishing the pancreatic islets, from exocrine tissue, and the background were created with the intention to train IsletNet neural network.

### Study participants

Nine invited experts from three independent centers participated in this pilot study, using IsletSwipe application on their mobile devices. They all had previous experience with islet counting in their respective centers. All the invited experts tested their drawing precision, validated their respective drawing tools, and tested their respective repeatabilities (more than presented in this study). Subsequently, they evaluated the user experience with IsletSwipe application. Eight invited experts from two centers proceeded to evaluate additional images to demonstrate two use case scenarios. On the other hand, the ground truth producing expert operated IsletSwipe web interface and analyzed the results obtained from the invited experts, but abstained from the above mentioned activities. Experts’ identities were anonymized throughout the study.

### Classification criteria

The islet micrographs are classified as good, borderline, or unacceptable (**Supplementary Fig. S1A**). The good quality means that the expert deems the image suitable for clinical islet counting without any hesitation. The borderline quality means that the micrograph can still be used for clinical counting, but with some reservation. The unacceptable quality means that the expert would not use it for clinical islet counting. The specific criteria are purposefully left to the individual experts who are encouraged to state their reasons in the Note before submitting the assessment.

Any arrow-marked tissue particle without contour was originally regarded as non-islet. When regarded by the invited expert as an islet, it should be classified as False (missing contour) and the full contour should be drawn or sketched.

### The usability assessment

A standardized and validated mixed-tone questionnaire of the System Usability Scale^27^ was administered separately and anonymously by an independent person to nine experts with prior experience with IsletSwipe, submitting over a hundred opinions including image classifications and islet contour drawing. The responses to 10 alternating tone questions were converted into numbers, summed up, and then converted to a mark using the curved grading scale according to a standard procedure.^27^

### Technical notes

IsletSwipe application is available for free from App Store and Google Play. It can run on iOS and Android smart phone or tablet. Experts interested to participate will receive an invitation e-mail with credentials from the corresponding author. Fingertip, rubber tip pen or stylus (Adonit),^28^ can be used for the drawing, according to personal preference.

## Results

### Platform design

**Figure 1.**
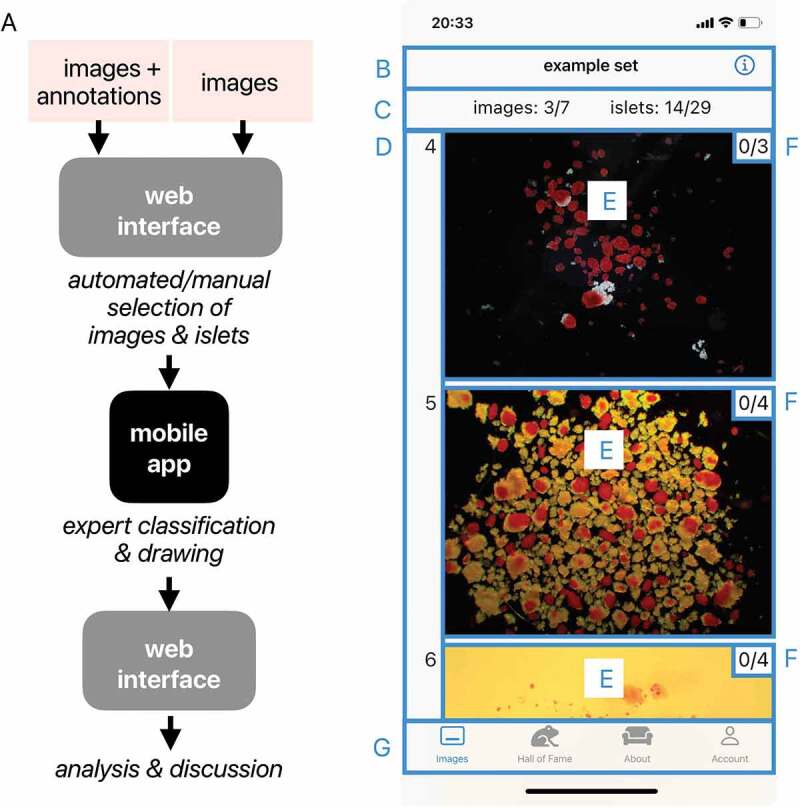
IsletSwipe platform. A: IsletSwipe platform components and the workflow. B-G: IsletSwipe application home screen. B: Title of the current set of images; C: Images and islets in the current set (the number if completed from the total number in the current set); D: Image identification numbers within the current set; E: The launching pads for individual images; F: The numbers of marked islets in the image (completed from total); G: The switching board.

The IsletSwipe platform consists of a web interface and a mobile application (Figure 1A). The web interface supports the management of images and expert users, including the assignment of sets of images to the experts. The images are presented to the experts in a mobile application which simultaneously provides the tools for expressing qualitative, graphical, and verbal opinions. The experts submit their opinions back to the web interface for further analysis. Results of the analysis are then shared among the participating experts.

The image upload to the web interface involves selecting the images and marking the islets for expert scrutiny. The uploaded images and marked islets are given identification numbers valid within the set. The selection process can be manual or automatic, depending on the purpose of the assessment (validation versus inquiry scenarios). The automated upload engine handles a zip file comprising the original images with annotations listed in a csv file with the respective pixel sizes. The engine selects a pre-set number of random images from the list and random islets from each image. The random islet selection proceeds separately within the 50 μm islet size categories^9^ calculated from the islet area^14^, with the adjustable size threshold, aiming to represent islets across the size categories. The selected islets are marked with arrows.

The user information is restricted to the name, e-mail, and institution, which are necessary for sending the access credentials and sharing the results of the subsequent analysis.

### Mobile app design

The application opens while loading the first three images from the set currently assigned to the logged user. The Home screen serves as an overview and the launching pad for the expert evaluation. It is divided into six sections (Figure 1B-G).

The title section at the top (Figure 1B) shows the name of the current set of images. It also contains the button ‘i’ for recalling an animated Tutorial. Below, there is a summary section (Figure 1C) which provides the expert a quick overview of the assessment progress, specifying the numbers of images and islets within the set (already submitted/out of the total). The section on the left margin (Figure 1D) shows the image identification numbers within the current set.

The Image previews in the center of the Home screen (Figure 1E) serve as the launching pads. Tapping opens the corresponding image for the expert’s assessment. The numbers in the Image preview the upper right corner (Figure 1F) represent the already evaluated/out of the total number of marked islets in the image.

The bottom section (Figure 1G) allows for switching between four screens of the application: Images, Hall of fame, About, and Account. The Account screen contains tools for troubleshooting. The Hall of Fame shows the expert’s ranking among the peers according to the numbers of submitted images within the current set.

### Mobile app functionalities

IsletSwipe supports the expression of three types of the expert opinions: qualitative (classification), graphical (drawing), and verbal (brief comments).

The microscopic quality of the images can be classified into three distinct categories: Good, Borderline, Unacceptable (**Supplementary Fig. S1A**, **Video 1**). The quality of the individual islet contours can be classified as True or False, with a possibility to refrain (**Supplementary Fig. S1B**). To avoid bias, the experts are encouraged to first consider the islet borders for themselves. For the context they can explore the entire picture and zoom in/out. Double click will return the islet under the scrutiny back to the screen center. Only after making an independent personal judgment, the islet contour under the scrutiny should be displayed/hidden as necessary, using the round Contour button (**Supplementary Fig. S1B, C**).

If the current contour is classified as True, the next islet shows up automatically. If the contour is classified as False, the drawing screen opens (**Supplementary Fig. S1C**) allowing the expert to correct it (**Video 2**). The expert can choose to indicate just the incorrect parts of the contour, cancel the object as non-islet, add separating lines between adjacent islets, or fully re-draw the islet contour. The islet position and zoom should be adjusted before dipping the pen into the ink-pot (Pencil icon). The move/zoom mode can be reactivated by the Magnifying glass icon. The Undo button allows for re-drawing the contour. The Skip button serves to refrain from classifying the present islet contour.

Note the Progress bar and the numbers above the image to keep track of the completed islets; use the Next and Back arrows to browse among the marked islets reviewing the current image.

Before Submitting the evaluation, experts are encouraged to leave comments by pressing the Note button (**Supplementary Fig. S1D**).

### Expert drawing accuracy

The positions of the islet separation lines and the embedded islet contours are expected to differ among experts. In order to quantify true differences, the intentions must be distinguished from random errors. Therefore, each expert’s accuracy needs to be experimentally established first. To this end, the experts were asked to reproduce red template lines that represented either the separation lines between adjacent islets or the full islet contours. Simplified pictures of islets were derived from original islet images, keeping the original dimensions and the pixel size.

**Figure 2.**
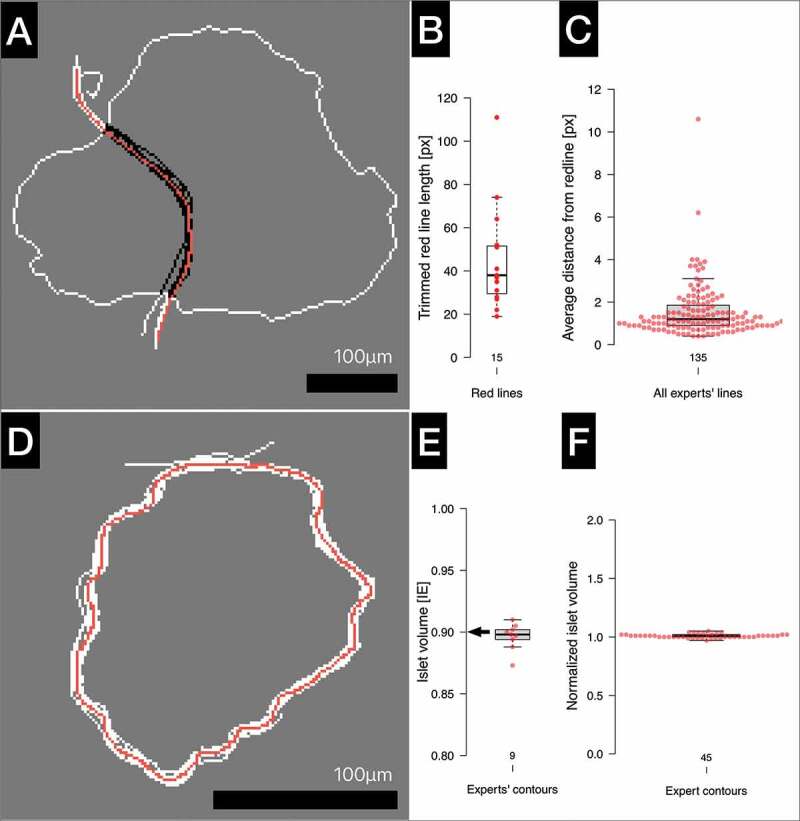
Experts’ drawing accuracy. A: A representation of two large adjacent islets (white contour) separated by a red line. Experts were tracing the red template line using IsletSwipe application without the drawing tool optimization. The original expert lines (white) were trimmed (black) before the measurement of the average distances from the trimmed (not shown) red template line. B: Box plot shows the distribution of the lengths of all 15 red lines after trimming. C: Box plot depicts all the average distances of the experts’ lines from the respective red lines. D: The full islet contour (red) was traced by the experts (white) using IsletSwipe application after the drawing tool optimization. E: Box plot compares the islet volumes derived from the experts’ lines to the volume derived from the red template line (arrow) for the islet shown in D. F: Box plot depicts all the experts’ islet volumes normalized to the respective red template islet volumes for all five islets.

Nine invited experts from three centers traced 15 red template lines separating adjacent islets (an example is shown in Figure 2A). The lengths of the 15 red template lines after trimming (see Methods) ranged between 19 and 111 pixels (Figure 2B). The average distance of the trimmed expert’s line (black) from the trimmed red template line was used to calculate expert’s drawing accuracy. The analysis of 135 experts’ lines (9 × 15) revealed that on average, the experts deviated from the red template line only by 1.5 pixels (median 1.2 px, Figure 2C). The accuracy of some experts was poor (>2 px, Figure 2C). As a result, these experts subsequently tested various drawing tools.

In the next and more challenging experiment, after the drawing tools optimization, the same experts were tracing the full islet contours of five islets with different shapes and sizes (volumes 0.9–7.6 IE). Most experts were now using a stylus rather than fingertip or a rubber pen tip.

An example of the red contour tracing by nine experts (white) is shown for a medium size islet in Figure 2D. The experts’ contours and the red template contours were converted into the islet volumes as depicted in Figure 2E.

All 45 experts’ contours (9 × 5) were eventually normalized to the corresponding volumes derived from the red template contours. The box plot analysis of the normalized volumes (Figure 2F) and the relative error (≤0.05, not shown) revealed very high accuracy among all 9 experts.

These data prove that IsletSwipe can support an accurate drawing by the islet experts when a personally suitable drawing tool is chosen. The drawing is easy, as demonstrated in a real-time **Video 3**. [Figure 2 near here]

#### Contour validation scenario

**Figure 3.**
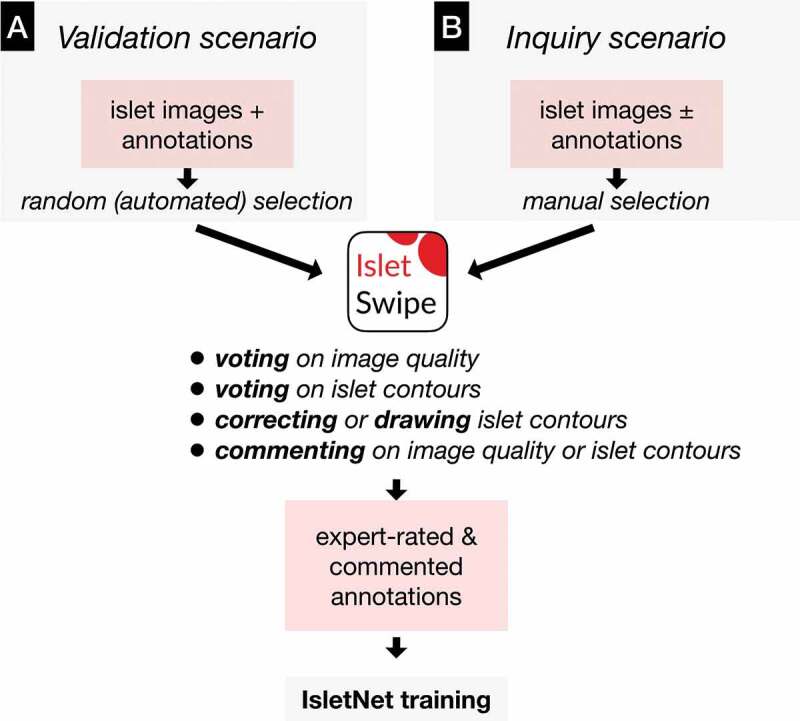
Two scenarios for IsletSwipe usage. A: Validation scenario allows the invited experts to validate islet contours generated by the ground truth producing expert or by IsletNet or from other sources; B: Inquiry scenario helps the ground truth generating expert in difficult cases.

The total of 19 islet images, the corresponding ground truth annotations (comprising 260 islets) and a csv file (containing the respective pixel size specifications) were uploaded to the IsletSwipe web interface. The islet size threshold was set to 50 µm and the intended number of random images to 8. The ensuing Validation-scenario set (Figure 3A) comprised eight randomly selected images with 33 randomly marked islets (26 were free and 7 embedded, the sizes ranged between 50 and 400 µm). There were 1–7 marked islets per image. Eight experts from two centers evaluated the set using IsletSwipe with a finger tip, a rubber pen tip, or a stylus. The mean time required for the evaluation was 16 min (±5 min, range 10–25 min).

**Figure 4.**
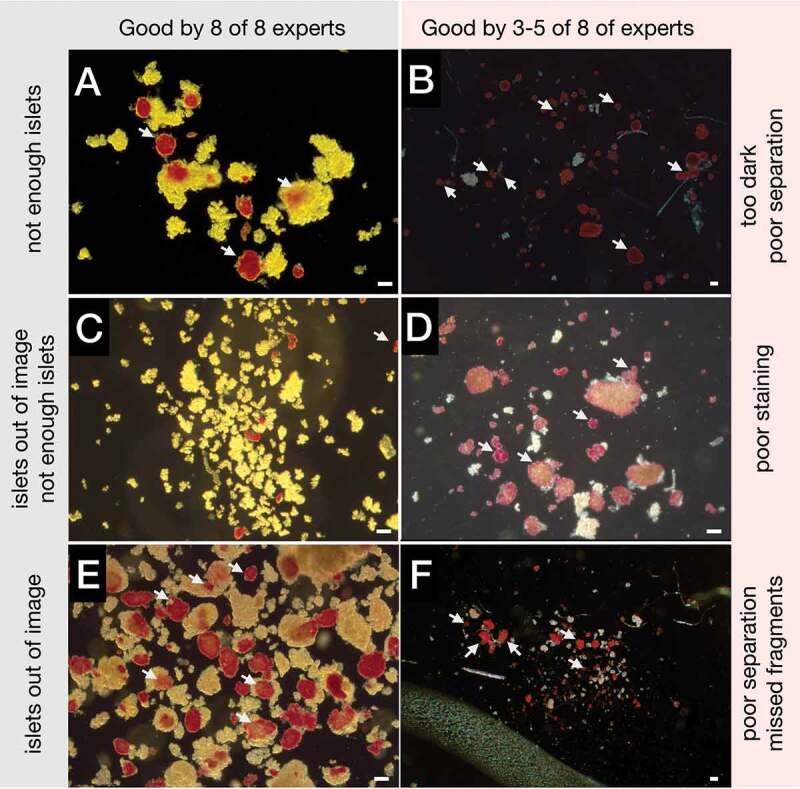
Validation scenario, the image quality classification and comments. A-C: Original images classified as good by all experts. D,E: Images classified as unacceptable by two experts. F: Images classified as unacceptable by a single expert. Arrows: islets automatically marked for the expert scrutiny; Bars: 100 µm.

The total of 64 (8 × 8) qualitative classifications of the islet micrographs were submitted. The subsequent analysis revealed that all experts agreed about the good microscopic quality of four images, but varied on the micrograph quality of the remaining four images (examples shown in Figure 4A-C vs. Figure 4D-F). The comments (Figure 4 sides) on the micrograph quality included incomplete sample view, image darkness, and poor islet staining. Some experts noted inadequacies in the ground truth contours such as poor islet separation, missing islet fragments, or missing the entire islets (not shown).

**Figure 5.**
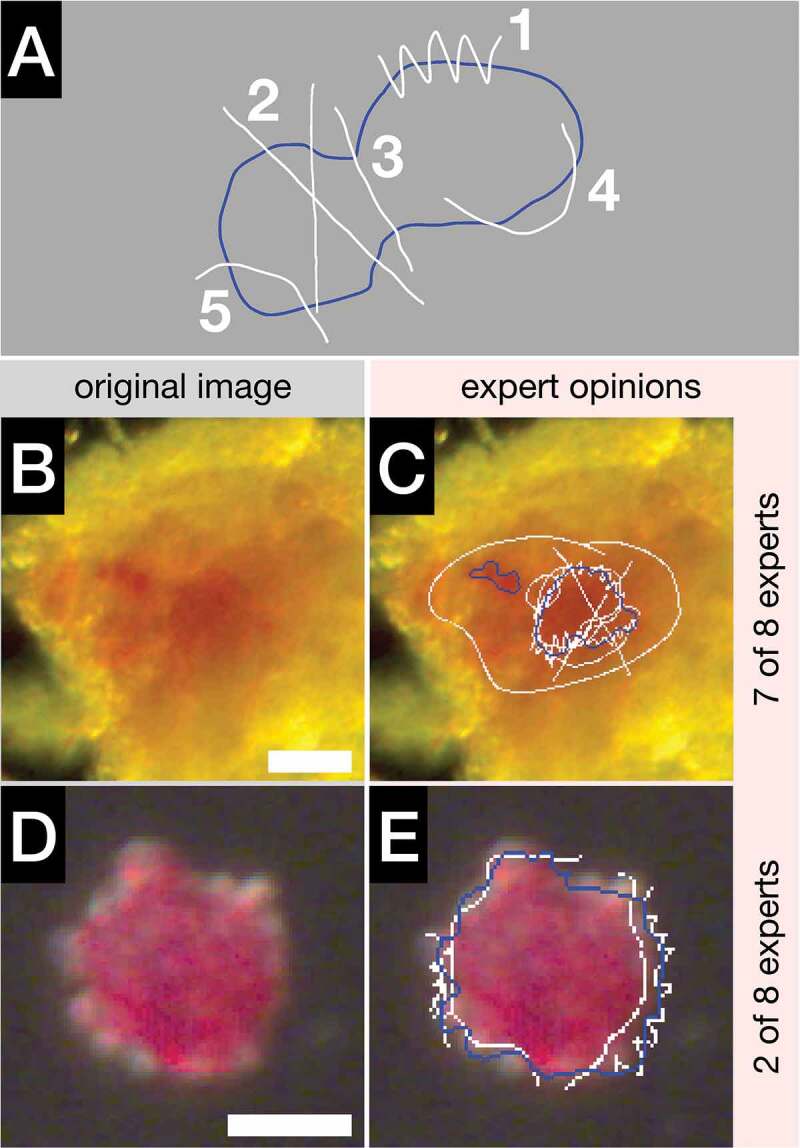
Expert annotations. A: Instructions for expert drawing: blue line: the ground truth annotation; white lines: expert annotations; 1: disagreement with a portion of the contour; 2: this is not an islet; 3: islet separation line; 4: include this part to the islet; 5: exclude this part from the islet. B: A detail of an embedded islet marked by arrow in the original image in Fig. 4A (right from the center). C: The same islet with the ground truth contour (blue) corrected by seven experts (white). D: A detail of a free islet marked by arrow in the original image in Fig. 4E (in the center). E: The same islet with the ground truth contour (blue) corrected by two experts (white). Bar: 50 µm.

The experts also submitted 69 graphical opinions to the ground truth annotations of 24 (out of 33) marked islets; 13 opinions related to 4 embedded islets and 56 opinions to 20 free islets (**Supplementary Figure S2A**). The experts contributed between 5 and 15 graphical opinions (median was 7.5). The disputed contours were fully redrawn or corrected by 1–8 experts, using the signs specified in the Legend (Figure 5A). Thirteen graphical changes to the contours of 4 (out of 7) embedded islets were drawn by 1–7 experts each. The diverse opinions ranged from “no islet,” to “a smaller islet,” to “a much larger islet” as demonstrated in an example shown in Figure 5C. The total of 56 graphical opinions on the annotations of 20 (out of 26) free islets were collected from 1 to 8 experts each. In 18 cases 2–7 experts added various numbers of islet separation lines to the contours of four tissue particles, splitting them into 2–4 islets (2 experts also corrected 2 contour outlines and one expert skipped one free islet for poor visibility). 36 corrected/re-drawn free islet contours were collected; 16 corrections were contributed by 3–5 experts on 4 islets and the remaining 20 corrections were collected from only 1–2 experts each on 14 islets (including the two mentioned above with added separation lines, yellow in **Supplementary Figure S2A**). Expert opinions ranged from inconsistent and minor to full contour re-drawing. One example of the contour correction by two experts in shown in Figure 5E. Finally, two free tissue particles on different images were not regarded as islets by two experts (magenta in **Supplementary Figure S2A**).

### Contour inquiry scenario

To demonstrate the Inquiry scenario (Figure 3B), the ground truth producing expert manually uploaded to IsletSwipe web interface a single image with a preliminary ground truth annotation, seeking the advice from the peers on 11 manually marked difficult objects regarded as 3 embedded islets and 8 fragments of exocrine tissue. Within 24 h, eight experts from two centers submitted their graphical opinions.

**Figure 6.**
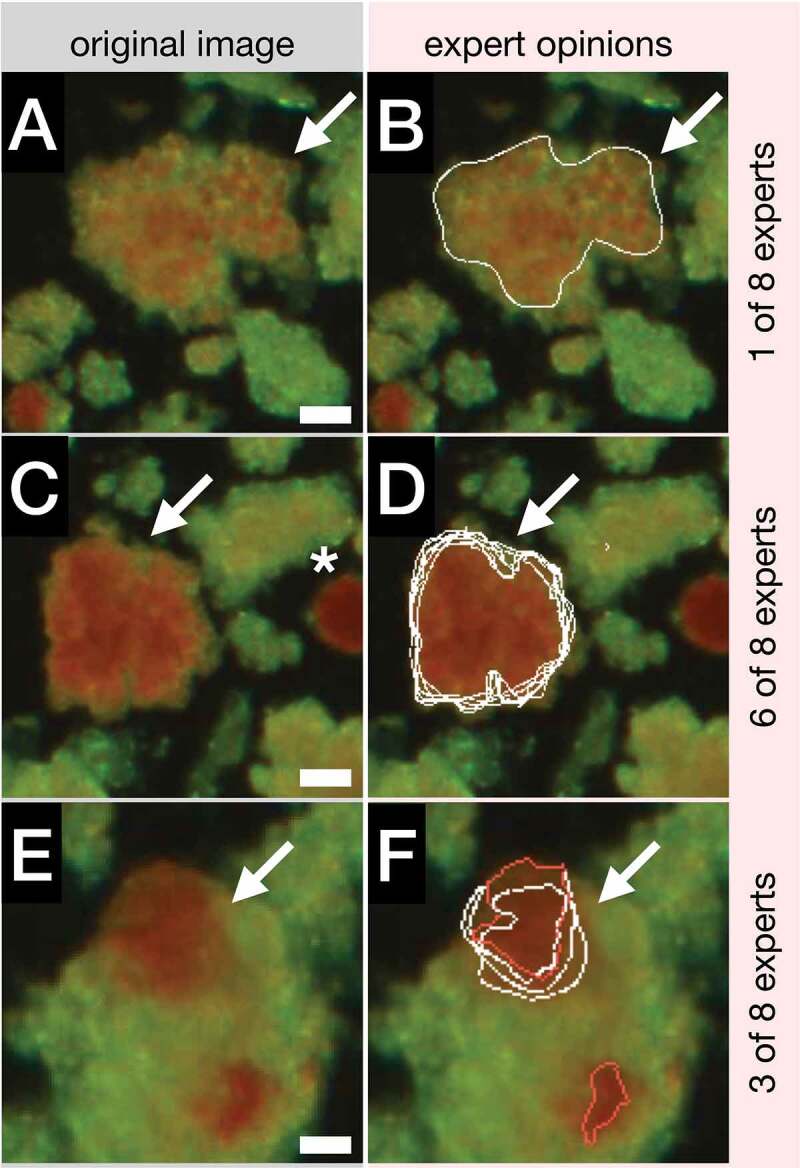
Inquiry scenario. A,C: Details of tissue particles regarded as non-islet tissue by the ground truth producing expert. B,D: Independent annotations by one and six invited experts, respectively. E: Detail of an embedded islet. F: The same islet with the ground truth contour (red) corrected by three invited experts (white). Arrows: tissue particles manually marked for the expert scrutiny. Asterisk: a well stained free islet for a reference. Bars: 100 µm.

The subsequent analysis revealed that there was no uniform agreement on the identity of the tissue particles originally classified as the exocrine tissue (**Supplementary Figure S2B**). Seven experts agreed that 3 particles were indeed an exocrine tissue, as originally annotated (Figure 6B). The remaining 5 particles were regarded as islets by 3–6 experts each (Figure 6D). While all the experts confirmed the identity of the embedded islets, three of them disagreed with the exact position of the ground truth contour but also among themselves (Figure 6F). One expert remained undecided on one tissue particle (turquoise, **Supplementary Figure S2B**).

### Experts’ reproducibility

**Figure 7.**
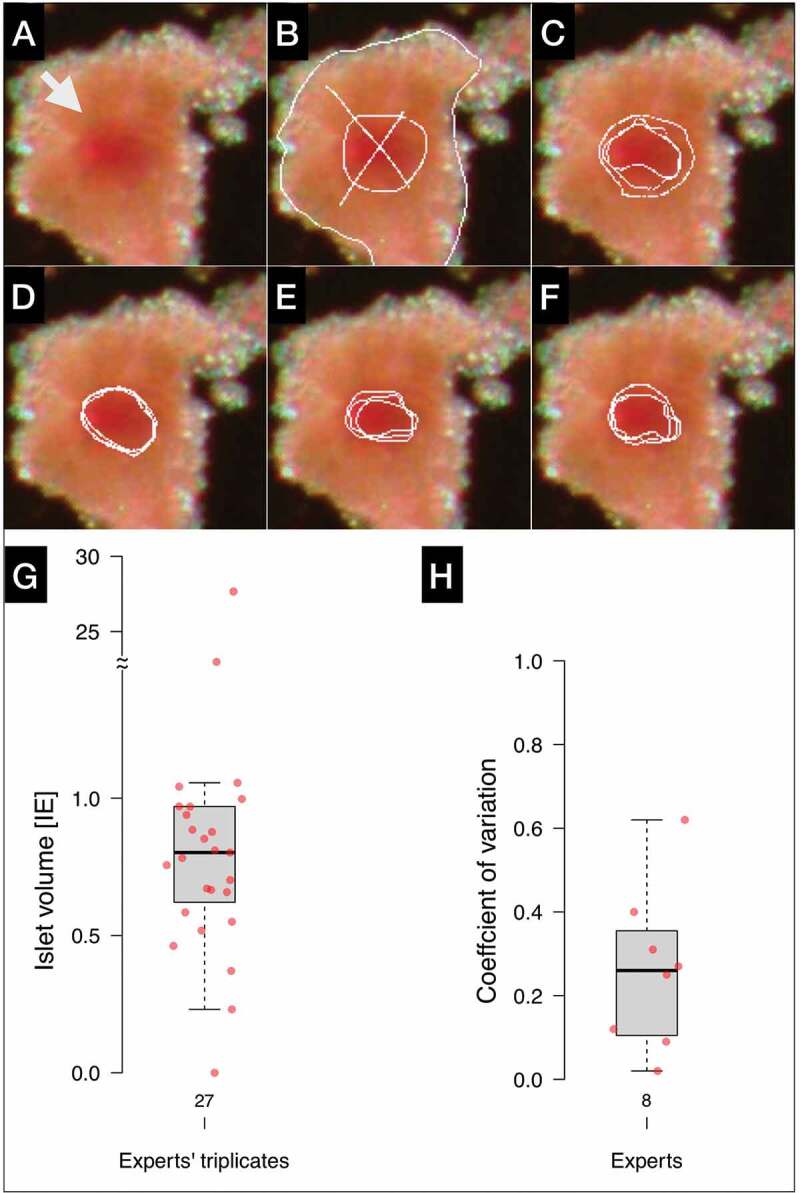
Experts’ reproducibility. A: An original image of an embedded islet. B-E: Examples of the triplicate contours by four invited experts, respectively. F: The triplicate contours by the ground truth producing expert. G: Box plot depicts all the islet volumes derived from the repeated experts’ contours; H: Box plot summarizes the individual experts’ reproducibility (excluding the outlier from G).

The capacity of IsletSwipe to test the consistency of the experts’ graphical opinions on the exact positions of the contours is demonstrated on an embedded islet depicted in Figure 7A. The islet was presented to nine experts from three centers at three occasions separated by at least one week of the weaning period.

The triplicate contours are shown for four experts from three centers (Figure 7B-E). For comparison, the triplicate lines of the ground truth producing expert are also shown (Figure 7F).

The triplicate contours (27 contours, 9 × 3) were converted into islet volumes (Figure 7G). Even when the least consistent expert (Figure 7B) was excluded, there still was a fivefold difference in the islet volume among the remaining experts (0.23–1.06 IE, Figure 7G). Next, the triplicate volumes from seven experts were averaged and the coefficients of variation were calculated (Figure 7H); the inconsistent expert was excluded from this calculation because one value of the triplicate was zero. The individual expert’s coefficients of variation ranged between 0.02 and 0.62, with the median 0.26.

For comparison, the coefficient of variation of the ground truth generating expert on this particular islet was 0.21 (the mean islet volume was 0.50 IE, range 0.43–0.59 IE, not shown).

### Mobile app usability

Nine experts from three independent centers rated their respective experiences with IsletSwipe application after using it for several months and evaluating dozens of images and islets (not presented here). The individual assessments ranged from A+ to B (in A+ to F scale) with the average rating A+ as detailed in Table 1. In addition to the standard questionnaire, critical comments and suggestions were obtained e.g.: “app itself is easy to use, but the act of drawing can be difficult;” “some of my older colleagues may struggle to understand the app without someone to show in person or via video call;” “enable using the best contour if several efforts were made.” While some experts were using fingertip all the time, others were willing to collaborate only after using the stylus. All experts preferred using a cell phone over a tablet.

**Table 1.** Assessment of IsletSwipe usability.

EXPERT	SUS	MARK
expert 1	95.0	A+
expert 2	90.0	A+
expert 3	87.0	A+
expert 4	87.5	A+
expert 5	87.5	A+
expert 6	85.0	A+
expert 7	85.0	A+
expert 8	82.5	A
expert 9	75.0	B
OVERALL	86.0	A+

*SUS, system usability score.

## Discussion

The procedure of clinical islet isolation is completed with the graft quality control step. The primary indicator of the graft quality is the total volume of pancreatic islets accompanied by the histogram of the islet size distribution and its purity. The work presented here fits into the larger effort to develop an objective, fully automated, accessible, and reliable tool for the islet counting, using deep-learning technology for extracting the useful information from microscopic images of the graft. Our web service IsletNet (https://isletnet.com) has been available since 2017.^21^ The neural network at its core was gradually trained using a wide range of images of dithizone-stained isolated pancreatic islets obtained from numerous centers across Europe and the USA.^22^

At present, IsletNet features the Validation tool “Simple comparison” available the participating centers comparing side-by side the islet count, volume, size histogram, and purity. This tool should satisfy the needs of individual centers. However, the aim of IsletNet is universal. Therefore, its satisfactory performance should be defined as such that generates the results falling within a range of expert opinions across the centers. IsletSwipe was developed to help collect such expert opinions (experts will evaluate the same set of images as the one used to validate IsletNet).

The novel IsletSwipe platform presented here was also developed to improve the quality of IsletNet training by providing a consensual guidance to the ground truth generating expert. While the islet boundaries seem mostly clear, the embedded islets, poorly stained islets, adjacent islets, mantled islets, or islet fragments impose a challenge with a risk of untoward subjectivity. Moreover, anecdotal evidence suggests, that even the boundaries of pure islets might be interpreted by some experts more generously than by others. Due to the lack of an effective communication tool at present, the degree and the areas of a consensus and a disagreement remain unknown. IsletSwipe is the first attempt to bridge this technological gap by providing the islet community an efficient platform for the expert discussion. Taking advantage of the current high-quality mobile communication devices, IsletSwipe brings the islet images and annotations simultaneously into the hands of interested individual experts from independent centers.

The aim of the platform is collecting not only the independent experts’ classifications of the islet contour quality, but also the exact opinions on the contour location. An experiment was therefore designed to measure the exact deviation between the intended and the actual line drawn by individual experts. On pictures of islet outlines, the experts were copying a predetermined lines representing the real islet shapes. Our results demonstrated that IsletSwipe application can indeed support a high accuracy drawing (median 1.2 px, Figure 2C). Therefore, the exact contours collected by IsletSwipe can be taken seriously, provided the author’s accuracy was objectively tested and an attention was paid to the choice of personally suitable drawing device (Figure 2F).

The main scenarios for IsleSwipe use were demonstrated in two small but instructive experiments (Figure 3). The hallmark of the Validation scenario is the unbiased selection of images and islets for the expert scrutiny. Here, eight experts evaluated the micrograph quality of eight randomized images and then evaluated/corrected the existent ground truth of 33 randomized islets. This scalable scenario (more experts, images, and islets) can be applied for an independent validation of islet contours from various sources (including the contours automatically generated by IsletNet). In this pilot study, the experts were given an opportunity to evaluate the contours generated by the ground truth producing expert (for IsletNet training).

On the other hand, the Inquiry scenario serves the ground truth generating expert when seeking specific assistance from the peers on the challenging cases encountered during the preparation for IsletNet training. This scenario is limited to a small number of manually selected images and islets, so that the peers can return their opinions in a short time period (here, eight experts returned their graphical opinions on eleven difficult islets within 24 h). In this concrete example, the practical outcome on the side of the ground truth generating expert was re-annotating three tissue particles as islets (e.g. Figure 6D) while keeping the original annotations for the rest (e.g. Figure 6A, Supplementary Figure S2B). This opinion exchange enabled the ground truth generating expert to annotate similar cases in the future with more confidence. Nevertheless, the contours of some embedded islets remained inconclusive (e.g. Figure 6F), calling for a more extensive IsletSwipe-mediated discussion involving experts from other centers.

The ground truth annotations as well as the automatic contours produced by IsletNet should be within the range of the expert opinions and thus the individual experts’ reproducibility. IsletSwipe revealed that the reproducibility of experts’ opinions varies and that a high individual consistency (CV = 0.02) can be achieved (Figure 7D,H).

The IsletSwipe-mediated opinion exchange presented here provided a valuable feedback to the ground truth generating expert. Also, the invited experts benefited by gaining an insight into and an influence over the IsletNet training. All the experts had an opportunity to refine their opinions in a wider context of other experts’ views (**Supplementary Figure S3**). After completing the evaluation, all the experts received cropped images (individual islets only) with their own contours. In addition, they received cropped images with the ground truth contour together with all the experts’ contours (anonymously). These images were intended as a base for independent thinking and making personal conclusions to be further discussed. This approach is illustrated on a tissue particle from the Validation scenario set (Figure 4A and Figure 5C). In **Supplementary Figure S3** the corresponding cropped images are depicted. This tissue particle was regarded as an embedded islet by the ground truth generating expert, while it was judged as a non-islet tissue by three experts, as a smaller islet by two experts, as a different islet by one expert, and as a larger islet by two experts. The experts’ drawings can be regarded as intentional, because they all successfully tested their accuracy (Figure 2F). Experts from more centers are welcome to IsletSwipe to help build a consensus.

The most experienced experts often have little time to spare on contour drawing, but their opinion matters. Addressing this issue, IsletSwipe supports a quick review and judgment (true or false) of a range of contours already produced by other experts, leaving the possibility to sketch proposed corrections (Figure 5A) or leaving a note. Such votes by the respected experts from different centers will help rank the existing opinions for the sake of a general discussion and the consensus building (**Supplement Figure S4**). Once consensus is reached, IsletNet can be effectively trained a general satisfaction.

Most experts are familiar only with the locally acquired images. IsletSwipe-mediated opinion exchange can inspire local improvements by two means. First, by presenting them images from other centers. Second, by providing an anonymous feedback on their own images.

Obtaining expert annotations is a notorious hurdle for the neural network training, mainly because the process is monotonous, tedious, and time-consuming. Often unexperienced nonprofessionals are paid to generate the ground truth data only after a brief instruction.^29–33^ Here we propose an innovative solution to the problem. IsletSwipe can be operated by real experts during brief idle time periods (e.g. commuting, waiting). The mobile device is almost always at hand and the work load is divided into very small packages taking a minute or two to complete. The progress bar helps quick orientation after a brake. After each package, the experts can enjoy a little award and see a personal ranking among the peers for friendly competition (Hall of Fame). These features should prevent expert’s fatigue.

The user experience with IsletSwipe application was assessed using a standard questionnaire completed by nine experts from three centers after an extensive experience with the application. IsletSwipe was rated as a user-friendly application (mark A+). Direct comments and suggestions were also collected. The most significant reservation was the necessity for one expert to re-install the application on several occasions in order to re-connect with the web interface (no expert evaluation was lost). The current pilot version of IsletSwipe serves as a proof of concept. If IsletSwipe finds its place in the islet community, a professional version will follow.

It might be tempting to think, that IsletNet should be trained only once and subsequently generate consistent results for all time in all centers. This assumes submitting training images by all centers at the same time and halting any progress in the image acquisition. In reality, the centers need time to consider joining IsletNet and even afterward they seek improvement by experimenting with the islet sample staining and the image acquisition techniques. The power of IsletNet deep-learning core is, that it can absorb newcomers and other sources of novelty, thus supporting the natural evolution in the field of islet counting and IsletSwipe will facilitate the process.

One future possibility is to connect IsletSwipe with IsletNet in a center-bound manner. After islet isolation the IsletNet-generated contours would automatically appear in the hands of the relevant local experts. This could become a quick undemanding way for a sustained local validation of IsletNet performance. A small number of randomly selected islets would be easy to complete in few minutes after isolation even by the colleague experts not actually present. This way, all interested members of the lab would be informed, enhancing the internal discussions. Should a drift from the standard staining occurred, it would be spotted on time and IsletNet would be re-trained on demand while keeping the good quality already achieved for other centers.

IsletSwipe could also support the training of new lab members with an appropriate set of educational images.

In conclusion, IsletSwipe is a promising, user-friendly and unique communication platform bridging the technological gap which hampered a more consensual IsletNet training. IsletSwipe has the potential to reveal discord among experts from independent centers while facilitating consensus formation, which in turn will be used to generate the ground truth for improved IsletNet training and validation. An undemanding IsletSwipe-mediated surveillance might help sustain a satisfactory IsletNet performance in the future. The standardized quantification of islets can help improve multicenter studies and clinical outcome of islet transplantation.

**Video 1**: Classification of an islet contour and drawing the islet separation line (using fingertip).

https://owncloud.ikem.cz/index.php/s/4ft8D83LQrlzQHv

**Video 2**: Classifying and correcting an islet contour with fingertip.

https://owncloud.ikem.cz/index.php/s/aJnkmjJs7uvzhBR

**Video 3**: Tracing red islet separation line with fingertip (for determination of expert’s accuracy).

https://owncloud.ikem.cz/index.php/s/DWOOFI8tBgem6E3

**Video 4**: Drawing embedded islet contours by two experts (using fingertip).

https://owncloud.ikem.cz/index.php/s/ljTGquKwYM4uNDG

## Supplementary Material

Supplemental Material
