# Supplementary figures and images for: IsletSwipe, a mobile platform for expert opinion exchange on islet graft images
Source: Islets. 2023 Mar 29;15(1):2189873. doi: 10.1080/19382014.2023.2189873 (PMC10064927; doi:10.1080/19382014.2023.2189873)

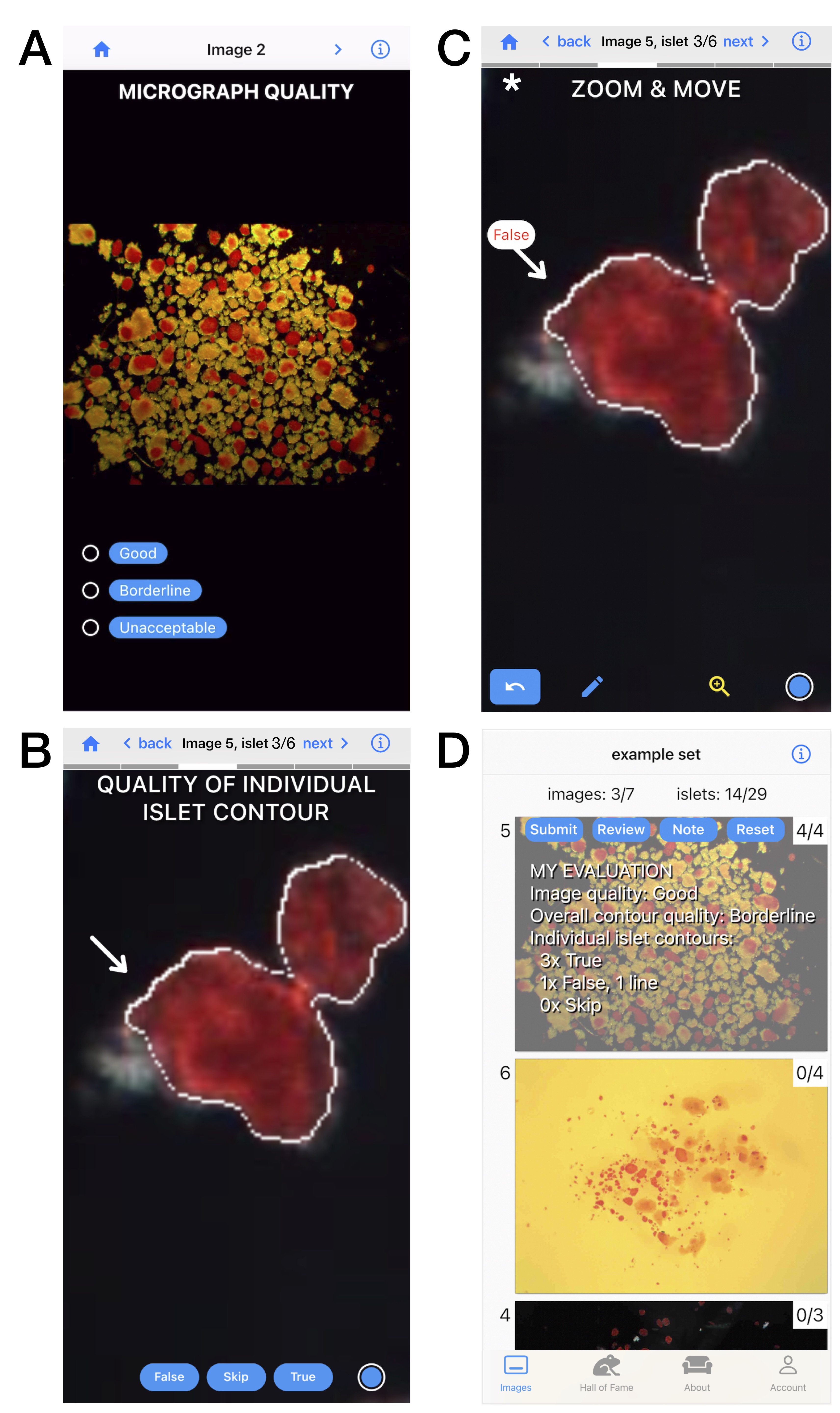

Supplement: Supplemental Material [file KISL_A_2189873_SM9878.zip › FigSuppl1_revised (1).jpg]

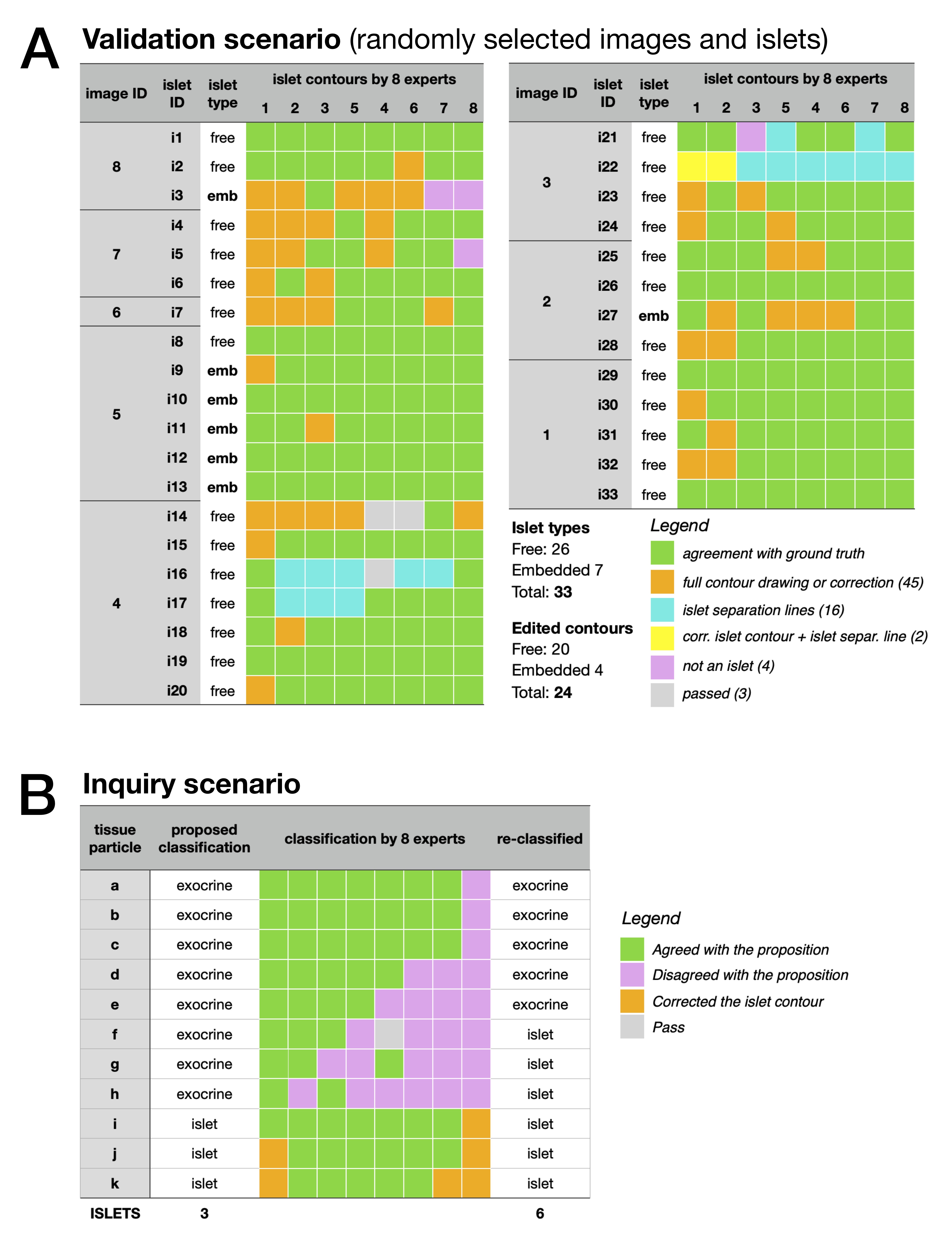

Supplement: Supplemental Material [file KISL_A_2189873_SM9878.zip › FigSuppl2_revised_v2.jpg]

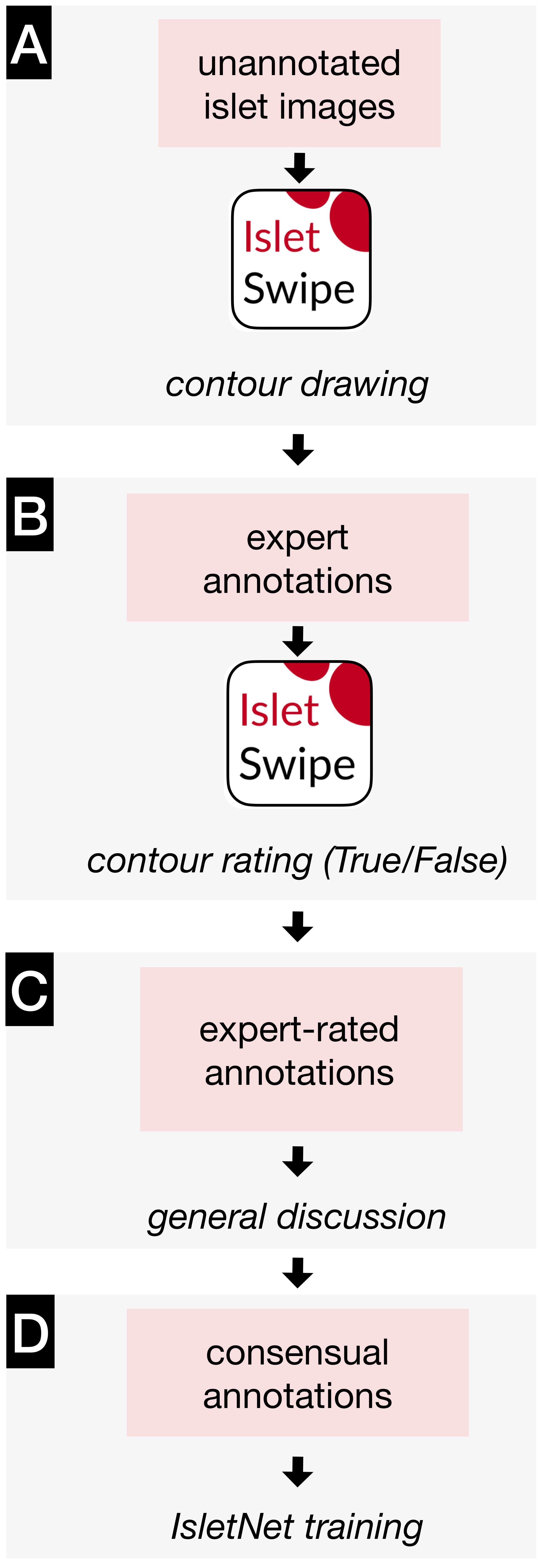

Supplement: Supplemental Material [file KISL_A_2189873_SM9878.zip › FigSuppl3_revised (1).jpg]
